# Deaths in the Medical Class

**Published:** 1848-03

**Authors:** 


DEATHS IN THE MEDICAL CLASS.

Two deaths have occurred in the session which is now closed. This is at the ratio of 1 in 133, for the year—two-thirds of one per cent. per annum. This rate of mortality is certainly low; and when we recollect, that almost all the students are from latitudes south of Louisville, down to the thirtieth degree, we must conclude that a winter residence in colder climates, is not so dangerous to southern constitutions as many have supposed. We have observed, in fact, that many young men improve in health and vigor, while sojourning here from October to March. When Dr. Cartwright lately visited Louisville, he was on a northern winter tour for the recovery of his health and strength—seriously broken down by his labors in the south, and he declared that both had been much improved.

